# Genome-Wide Association Studies for Cerebrospinal Fluid Soluble TREM2 in Alzheimer’s Disease

**DOI:** 10.3389/fnagi.2019.00297

**Published:** 2019-10-25

**Authors:** Changan Liu, Jun Yu

**Affiliations:** ^1^Department of Mathematics, University of Houston, Houston, TX, United States; ^2^Department of Medicine and Therapeutics, Faculty of Medicine, The Chinese University of Hong Kong, Hong Kong, China

**Keywords:** Alzheimer’s disease, soluble TREM2, cerebrospinal fluid, GWAS, SNP, immune

## Abstract

Alzheimer’s disease (AD) is the most common form of dementia. Rare variants in triggering receptor expressed on myeloid cells 2 (TREM2) have been identified as risk factors for AD. Soluble TREM2 (sTREM2) in the cerebrospinal fluid (CSF) is a potential and novel biomarker of neuroinflammation implicated in the onset and progression of AD. To explore the roles of CSF sTREM2 on the pathogenesis of AD, we performed genome-wide association studies (GWAS) by using the data from Alzheimer’s Disease Neuroimaging Initiative (ADNI). We found CSF sTREM2 levels were elevated with the disease stages, but there was no significant difference between that of AD patients and normal participants. CSF sTREM2 was positively correlated with CSF total tau and phosphorylated-tau levels (ρ > 0.35, *p* < 1e-06; ρ > 0.32, *p* < 1e-05, respectively) for all disease states. We identified the most significant CSF sTREM2 related locus was rs7232 (FDR = 3.01e-08), a missense variant in MS4A6A gene of chromosome 11. Moreover, we also detected rs7232 was highly associated with MS4A6A gene expression (FDR = 1.37e-18). In addition, our pathway analysis for our significant GWAS results showed that biological processes for regulation of viruses and immune response were highly overrepresented or enriched. Our study suggests that CSF sTREM2 plays an informative role in AD progression. Moreover, CSF sTREM2 and AD is highly related to viral infections and immune response.

## Introduction

The most common neurodegenerative disorder, Alzheimer’s disease (AD), is characterized by accumulation of amyloid-β peptide (Aβ) in senile plaques and hyperphosphorylated tau protein in neurofibrillary tangles (NFT) in brain, neuroinflammation, and progressive decline in cognition and memory loss finally (Price and Sisodia, 1998; Mukaetova-Ladinska et al., 2015). Although AD is a complex, multigenic and multifactorial disorder, most genetic, physiological and pathological studies suggest that the balance between production and clearance of Aβ contributes to its development (Hardy and Higgins, 1992). Microglia are the brain-resident effective phagocytes for the uptake and proteolytic clearance of both soluble Aβ oligomers and insoluble Aβ fibrils (Lee and Landreth, 2010). Many studies have detected that microglia surrounded Aβ plaques in the brain of AD patients (Perlmutter et al., 1990) and AD mouse models (Stalder et al., 1999) to reduce their sizes and subsequent toxicity (Condello et al., 2015). The protein triggering receptor expressed on myeloid cells 2 (TREM2) is an innate immune receptor expressed on microglia and on myeloid cells outside the brain (Schmid et al., 2002). It plays crucial roles in microglial phagocytosis of apoptotic neurons, damaged myelin, and Aβ plaques (Jay et al., 2017). In addition, TREM2 regulates microglial proliferation, survival (Zheng et al., 2017), cytokine release (Zhong et al., 2015), and the accumulation around Aβ plaques (Yuan et al., 2016). Several studies reported that the rare TREM2 mutation, arginine 47 to histidine (p.R47H, rs75932628) substitution in the extracellular immunoglobulin domain, significantly increases the risk for AD with odds ratios similar to those of carrying an apolipoprotein E (APOE) ε4 allele (Guerreiro et al., 2013; Jonsson et al., 2013), which is present in about half of late-onset AD (LOAD) patients and has been convincingly demonstrated to affect its risk (Bertram et al., 2010).

TREM2 protein undergoes proteolytic cleavage by ADAM proteases, releasing its ectodomain into the extracellular space as the form of soluble TREM2 (sTREM2) (Wunderlich et al., 2013), which is abundantly detected in human cerebrospinal fluid (CSF) and plasma. Although elevated CSF sTREM2 levels were initially reported in neuroinflammatory conditions such as multiple sclerosis (Piccio et al., 2008), exploring the relationship between CSF sTREM2 and other biomarkers of AD has recently become of great interest. Recent study reported that CSF sTREM2 exerts functional roles in microglia by promoting inflammatory responses and shielding them from apoptosis (Zhong et al., 2017). Furthermore, an AD mouse model study demonstrates a protective role of sTREM2 against amyloid pathology and related toxicity (Zhong et al., 2019). However, the roles of CSF sTREM2 on the pathogenesis of AD remain unclear. To gain insight into the mechanism of AD development according to CSF sTREM2, in this study, we analyzed the correlation of CSF sTREM2 levels with AD status and other important CSF and clinical biomarkers from Alzheimer’s Disease Neuroimaging Initiative (ADNI) cohort. Moreover, we performed genome-wide association studies (GWAS) to identify novel variants and genes associated with sTREM2 level and AD by using CSF sTREM2 and gene expression data as phenotypes. Additionally, we implemented pathway analysis for sTREM2 related genes to get a better understanding of AD pathology.

## Materials and Methods

Data used in this study were obtained from the ADNI database^1^. ADNI was launched in 2003 by the National Institute on Aging (NIA), the National Institute of Biomedical Imaging and Bioengineering (NIBIB), the Food and Drug Administration (FDA), and by private pharmaceutical companies and non-profit organizations, as a public-private partnership. The principal investigator of ADNI is Michael W. Weiner, MD. The primary goal of ADNI has been to test whether serial magnetic resonance imaging (MRI), positron emission tomography (PET), biological markers, and clinical and neuropsychological assessment can be combined together to measure the progression of AD.

We applied for and were granted permission to obtain data from the ADNI cohort^2^ for performing the analyses described in this paper.

### Subjects

In this work, analyses were restricted to ADNI subjects with CSF sTREM2 data available. The study sample (*N* = 1001) included 224 healthy normal (NL), 72 significant memory concern (SMC), 234 early mild cognitive impairment (EMCI), 277 late mild cognitive impairment (LMCI), and 194 AD participants. Table 1 shows selected demographic and clinical characteristics of these subjects at baseline.

**TABLE 1:** Selected demographic and clinical characteristics of 1001 ADNI participants at baseline.

	**NL**	**SMC**	**EMCI**	**LMCI**	**AD**
Number of subjects	224(22.4%)	72(7.2%)	234(23.3%)	277(27.7%)	194(19.4%)
Age (years)	74.34 (5.96)	71.74 (5.57)	71.06 (7.3)	73.04 (7.6)	74.53 (8.38)
Number of women	108(48.2%)	41(56.9%)	99(42.3%)	110(39.7%)	81(41.8%)
Education (years)	16.26 (2.73)	16.85 (2.44)	15.78 (2.63)	16.23 (2.91)	15.42 (2.81)
APOE ε4 allele present	55(24.6%)	25(34.7%)	105(44.9%)	155(56%)	130(67%)
CDR-SB	0.03 (0.12)	0.07 (0.17)	1.34 (0.79)	1.67 (0.95)	4.47 (1.59)
MMSE	29.05 (1.18)	29.13 (1.02)	28.34 (1.6)	27.22 (1.81)	23.31 (2)

*CDR-SB: clinical dementia rating-sum of boxes. MMSE: mini-mental status examination. Data in the table are illustrated in the form of “number (%)” or “mean (standard deviation).”*

### CSF and Clinical Biomarkers

Cerebrospinal fluid sTREM2 measurements were done with the ELISA protocol previously established by the Haass’ group with minor changes. The assay is based on the MSD platform and it is comprehensively described in their previous publications (Suárez-Calvet et al., 2016a, b). The CSF levels of amyloid-β 1-42 peptide (Aβ_42_), total tau (tau) and tau phosphorylated at the threonine 181 (p-tau) were determined using the fully automated Roche Elecsys immunoassay platform (Seibyl et al., 2017). The 13-item version of the Alzheimer’s Disease Assessment Scale-Cognitive subscale (ADAS13) was developed to measure memory and cognition for patients with mild to moderate AD (Podhorna et al., 2016). ADAS13 scores were automatically calculated on the electronic case report form based on item level data entered. Semi-automated hippocampal volumetry was carried out using a commercially available high dimensional brain mapping tool (Medtronic Surgical Navigation Technologies (SNT), Louisville, CO, United States) for MRI data of subjects, that has previously been validated and compared to manual tracing of the hippocampus (Hsu et al., 2002).

### Genotyping Data

The single-nucleotide polymorphisms (SNPs) data of ADNI-1, ADNI-GO, and ADNI-2 cohorts were collected from either the Illumina 2.5-M array or the Illumina OmniQuad array (Saykin et al., 2010; Shen et al., 2010). The SNPs shown in both arrays were used for the following analyses.

Quality control (QC) analysis was conducted by using R package snpStats (Clayton, 2012) in R software (R Core Team, 2013). In the QC, we excluded any SNPs that did not meet any of the following criteria: (1) SNPs on chromosome 1-22; (2) call rate per SNP > 95%; (3) Hardy-Weinberg equilibrium (HWE) test of *p*-value >10^–6^ (absolute value of *z*-score <4.753424). After QC analysis, 2,379,855 SNPs remained for the subsequent analyses.

### Gene Expression Data

Gene expression profiles of peripheral blood samples from ADNI participants were performed at Bristol-Myers Squibb (BMS) laboratories. The Affymetrix Human Genome U219 Array^3^ was used for expression profiling, which contains 530,467 probes for 49,293 transcripts. Raw expression values obtained directly from CEL files were pre-processed using the Robust Multi-chip Average (RMA) normalization method (Vawter et al., 2004).

### Statistical and Genetic Analyses

The CSF sTREM2 levels for different disease stages and their correlation with other CSF and clinical biomarkers were determined by using R software. To identify the association between SNPs and CSF sTREM2 levels, we performed (quantitative trait locus) QTL analysis using the R package Matrix expression quantitative trait loci (eQTL) (Shabalin, 2012). In this analysis, age, gender (1 for male and 2 for female) and diagnosis (1 for NL, 2 for SMC, 3 for EMCI, 4 for LMCI, and 5 for AD) at baseline were considered as covariates. Manhattan plots of QTL results were generated using the R package qqman (Turner, 2014). The regional plot for QTL results was obtained from LocusZoom^4^ (Pruim et al., 2010). The linkage disequilibrium (LD) plot was generated through R package gaston. The association between significant SNPs (FDR < 0.05) of our QTL results and gene expression data was detected by performing eQTL analysis. eQTL analysis was also conducted using the R package Matrix eQTL for the filtered SNPs, with the same covariates as our QTL analysis. Here, we considered both *cis-*eQTL analysis (local, distance < 1Mb) and *trans-*eQTL analysis (distant, distance ≥ 1Mb or the SNPs even locate on different chromosomes).

### Pathway Analyses

For the significant genes (FDR < 0.05) of SNP-gene pairs form our eQTL results, gene ontology (GO) analyses were performed using the Protein Analysis Through Evolutionary Relationships (PANTHER) statistical over-representation test v14.1^5^, which used data from the Gene Ontology Consortium (GOC)^6^. PANTHER utilizes a binomial distribution test to calculate overrepresentation of candidate genes, relative to background, for different GO terms (Mi et al., 2019). Additionally, we also conducted gene set enrichment analysis (GSEA) for these genes through the software GSEA Desktop v3.0^7^ (Subramanian et al., 2005). Here, we applied R package limma (Ritchie et al., 2015) to make the pre-ranked gene list as the input for GSEA, according to the gene expression data for NL and AD subjects.

## Results

### Correlations of sTREM2 With AD Biomarkers

**FIGURE 1:**
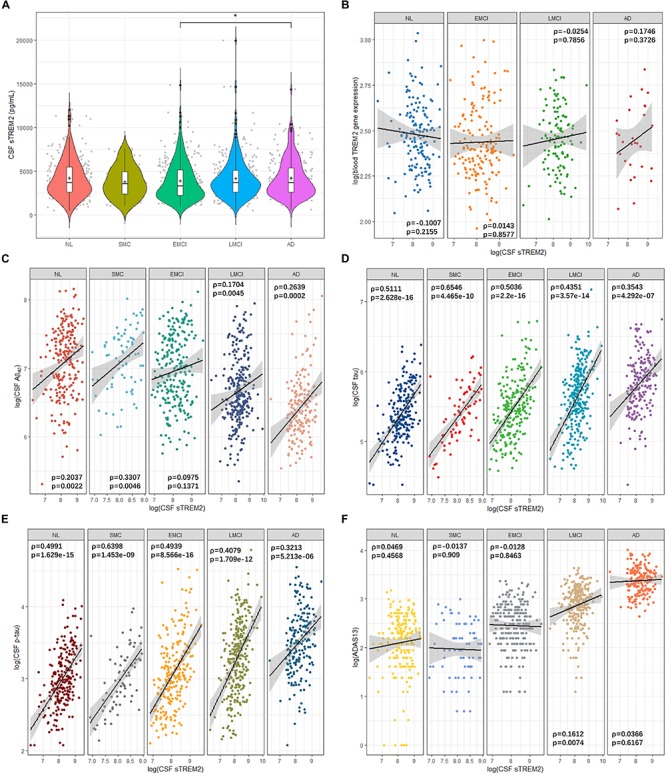
Cerebrospinal fluid sTREM2 levels in the five clinical disease stages and the correlation between sTREM2 and other AD biomarkers. **(A)** Violin plots with boxplots for the comparison of CSF sTREM2 levels in disease states. Statistical significance was determined by Wilcoxon rank sum test. +: mean. ^∗^*p* < 0.05. The correlation plots between log transformed CSF sTREM2 and **(B)** log transformed TREM2 gene expression in blood samples, **(C)** log transformed CSF Aβ_42_, **(D)** log transformed CSF tau, **(E)** log transformed CSF p-tau, and **(F)** log transformed ADAS13 scores for each group. Black straight lines are the regression lines. Shaded areas around regression lines represent the pointwise 95% confidence intervals (CI). ρ: Spearman’s rank correlation coefficient (rho).

In ADNI cohort, we saw that CSF sTREM2 levels increased with the disease progression from SMC group to AD group (mean: 3801.212, 3891.837, 4176.13, and 4216.861 pg/mL for SMC, EMCI, LMCI, and AD subjects, respectively; Figure 1A). However, only the difference between EMCI state and AD state was significant (*p* < 0.05; Figure 1A). Though the mean CSF sTREM2 levels for AD patients was a little higher than that of normal subjects (4206.102 pg/mL), there was no significant difference.

Levels of sTREM2 in the CSF did not correlate with TREM2 gene expression in the blood samples (Spearman’s ρ from −0.1007 to 0.1746, *p* > 0.05; Figure 1B). Next, we investigated the correlation of its CSF levels with other AD highly associated CSF and clinical biomarkers to explore its role in AD pathology. CSF sTREM2 levels showed highest correlation with CSF Aβ_42_ in SMC group (ρ = 0.3307, *p* = 0.0046; Figure 1C), followed by AD group (ρ = 0.2639, *p* = 0.0002; Figure 1C). The overall correlation between CSF sTREM2 and CSF tau were high for all the five disease states (ρ from 0.3543 to 0.6546, *p* < 1e-06; Figure 1D). SMC group showed the highest correlation (ρ = 0.6546, *p* = 4.465e-10) while AD group had the lowest one (ρ = 0.3543, *p* = 4.292e-07; Figure 1D). Similarly, CSF sTREM2 also correlated with CSF p-tau for all the five categories (ρ from 0.3213 to 0.6398, *p* < 1e-05; Figure 1E). Same as the case for CSF tau, the correlation of SMC group ranked first while that of AD was the smallest. For ADAS13 scores, the correlation between them and CSF sTREM2 were low generally (ρ from −0.0137 to 0.1612; Figure 1F). Moreover, there was no strong correlation between CSF sTREM2 levels and hippocampus volumes (ρ from −0.1483 to 0.0082, *p* > 0.05; Supplementary Figure S1).

### GWAS of sTREM2 Levels in CSF

**FIGURE 2:**
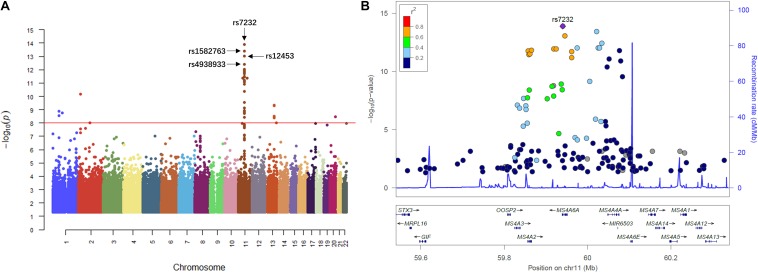
Manhattan plot and regional plot of the results from QTL analysis for CSF sTREM2 levels. **(A)** Manhattan plot of –log_10_ (*p*-value) from the results of QTL analysis. **(B)** Regional plot for the most significant SNP rs7232 identified by the QTL analysis. The *r*^2^ measures the linkage disequilibrium of each SNP with the most significant SNP rs7232 according to hg19/1000 Genomes Nov 2014 AMR population. Points with gray color indicates that their *r*^2^ values were not available in the reference genome.

We then performed QTL analysis to study the association between genotype (SNPs) and CSF sTREM2 levels. According to our analysis, the significant loci concentrated in chromosome 11 (Figure 2A). The most significant one was rs7232 (*p* = 1.32e-14, FDR = 3.01e-08; Table 2 and Figure 2), a missense variant in MS4A6A gene locus. The regional association plot of rs7232 is shown in Figure 2B. The second most significant SNP was rs1582763 (*p* = 3.85e-14, FDR = 4.41e-08; Table 2), which locates near the MS4A4E gene. The top 10 most significant SNPs were all located on chromosome 11. Moreover, they were either within or near to MS4A gene family (Table 2). Besides identified SNPs on chromosome 11, there were also SNPs showed significant association on other chromosomes, such as rs3799468 (*p* = 4.10e-12, FDR = 2.85e-07) located in MAP7 gene on chromosome 6, rs181768270 (*p* = 4.19e-12, FDR = 2.85e-07) proximal to gene RNF187 on chromosome 1, and rs116087048 (*p* = 4.19e-12, FDR = 2.85e-07; Supplementary Table S1) within the gene ASIC4 on chromosome 2. The list for all identified SNPs with *p* < 0.05 from our QTL analysis can be found in Supplementary Table S1.

**TABLE 2:** Top 10 most significant SNPs identified by the QTL analysis for CSF sTREM2.

**SNP**	**CHR**	**Gene**	**Function**	**Statistic**	***p*-value**	**FDR**	**Beta**
rs7232	11	MS4A6A	Missense	−7.9399	1.32e-14	3.01e-08	−1076.5072
rs1582763	11	MS4A4E^∗^	None	−7.7891	3.85e-14	4.41e-08	−109.9662
rs12453	11	MS4A6A	Synonymous	−0.6665	9.11e-14	6.95e-08	−1045.7299
rs4938933	11	MS4A4A^∗^	None	−7.4593	3.81e-13	2.18e-07	−1034.9755
rs6591559	11	MS4A4E^∗^	None	−7.3261	9.42e-13	2.85e-07	−1008.564
rs1530914	11	MS4A4E^∗^	None	−7.3261	9.42e-13	2.85e-07	−1008.564
rs7929589	11	MS4A4E	Intronic	−7.3183	9.92e-13	2.85e-07	−1009.3449
rs11230160	11	MS4A6A^∗^	None	−7.2926	1.18e-12	2.85e-07	−971.0224
rs920573	11	MS4A6A^∗^	None	−7.2926	1.18e-12	2.85e-07	−971.0224
rs2847655	11	MS4A2	3′-UTR	−7.2577	1.49e-12	2.85e-07	−959.4016

*CHR: chromosome. ^∗^Nearest gene proximal to the SNP. Without this symbol means the SNP is located within the corresponding gene. Statistic: *t*-statistic of *T*-test. FDR: false discovery rate estimated with Benjamini–Hochberg procedure. Beta: effect size (slope coefficient of the linear regression model) estimate.*

**FIGURE 3:**
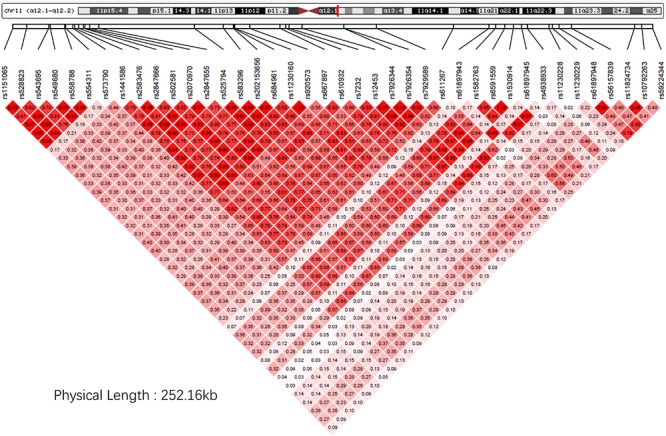
The linkage disequilibrium pattern for the identified SNPs close to rs7232 in our study. Here the LD measure is *r*^2^.

Next, for exploring the interactions among the identified significant SNPs on chromosome 11, we selected the SNPs with FDR < 0.05 and located within ±1Mb from rs7232. 40 SNPs including rs7232 remained for the analysis. The LD pattern for these 40 SNPs is illustrated in Figure 3. rs7232 showed strong LD with all the rest of top 10 identified most significant SNPs (Figure 3). Among them, the weakest LD occurred between rs7232 and rs1582763 (*r*^2^ = 0.68), while the LD between rs7232 and rs12453 was the strongest (*r*^2^ = 0.83; Figure 3). The strong LD between rs7232 and rs12453 may be due to that both of them locate in same gene MS4A6A. The LD of the SNP pairs rs1151065-rs558788, rs11230160-rs920573, and rs6591559-rs1530914 were extremely high (*r*^2^ = 1.00 for all the three SNP pairs; Figure 3).

For we wanted to study how the SNPs associated with CSF sTREM2 levels regulated gene expression, we chose the SNPs with FDR < 0.05 identified by our QTL analysis for the following eQTL analysis. There were 240 SNPs left for this analysis. In our *cis-*eQTL analysis (local eQTL analysis, the distance between SNPs and associated genes is less than 1Mb), the top 10 identified most significant SNPs were all located on chromosome 11, and they all associated with MS4A6A gene (Table 3). rs12453 ranked first (*p* = 9.02e-23, FDR = 8.14e-19; Table 3) in the results. Another SNP within MS4A6A gene, rs7232, showed the third most significant association (*p* = 4.57e-22, FDR = 1.37e-18) with it. Furthermore, rs1582763 was also significantly associated with MS4A6A gene expression (*p* = 3.58e-17, FDR = 1.9e-14; Supplementary Table S2). For the results of *trans-*eQTL analysis (distant eQTL analysis, the distance between SNPs and associated genes is bigger than 1Mb, or they even locate on different chromosomes), the association between rs113239743 and CPT1C gene was the most significant (*p* = 6.53e-44, FDR = 7.73e-37; Table 4). Moreover, rs113239743 also showed significant association with TESMIN gene (*p* = 3.91e-30, FDR = 9.27e-24), and CCND2 gene (*p* = 2.43e-25, FDR = 2.39e-19). The results for all identified SNP-gene pairs with *p* < 0.05 from our *cis-*eQTL analysis and those with FDR < 0.05 from *trans-*eQTL analysis are demonstrated in Supplementary Tables S2, S3, respectively.

**TABLE 3:** Top 10 most significant SNP-gene pairs identified by our *cis-*eQTL analysis.

**SNP**	**CHR**	**Associated gene**	**Statistic**	***p*-value**	**FDR**	**Beta**
rs12453	11	MS4A6A	10.1568	9.02e-23	8.14e-19	0.132
rs7926354	11	MS4A6A	10.0141	3.24e-22	1.37e-18	0.1298
rs7232	11	MS4A6A	9.9752	4.57e-22	1.37e-18	0.1302
rs7926344	11	MS4A6A	9.7366	3.73e-21	8.40e-18	0.1269
rs4938933	11	MS4A6A	9.6392	8.68e-21	1.50e-17	0.1272
rs7929589	11	MS4A6A	9.6231	9.97e-21	1.50e-17	0.1259
rs1530914	11	MS4A6A	9.5625	1.68e-20	2.17e-17	0.1255
rs6591559	11	MS4A6A	9.5333	2.16e-20	2.44e-17	0.1253
rs610932	11	MS4A6A	9.3276	1.24e-19	1.25e-16	0.1222
rs611267	11	MS4A6A	8.997	1.95e-18	1.76e-15	0.118

**TABLE 4:** Top 10 most significant SNP-gene pairs identified by our *trans-*eQTL analysis.

**SNP**	**CHR**	**Associated gene**	**Statistic**	***p*-value**	**FDR**	**Beta**
rs113239743	1	CPT1C on chr11	−14.8672	6.53e-44	7.73e-37	−2.5852
rs2971627	2	LUM on chr12	−12.7471	9.03e-34	2.67e-27	−2.292
rs2911645	2	LUM on chr12	−12.7471	9.03e-34	2.67e-27	−2.292
rs113239743	1	TESMIN on chr11	−11.9332	3.91e-30	9.27e-24	−2.5031
rs115619982	17	OTOF on chr2	−11.3008	2.03e-27	3.01e-21	−3.056
rs114191746	2	CRISP2 on chr6	−11.2371	3.77e-27	4.96e-21	−1.6717
rs115904095	2	CRISP2 on chr6	−10.8452	1.59e-25	1.71e-19	−1.6216
rs113239743	1	CCND2 on chr12	−10.8003	2.43e-25	2.39e-19	−0.5796
rs2814778	1	SOS1 on chr2	−10.6572	9.25e-25	5.40e-19	−0.5519
rs76465000	12	CCL2 on chr17	−10.6327	1.16e-24	5.40e-19	−4.6426

### Pathway Studies

For the SNP-gene pairs identified from both *cis-*eQTL and *trans-*eQTL analysis for CSF sTREM2, we chose the genes from the results with FDR < 0.05 for the following pathway analysis. We found 4295 qualified genes. Firstly, we performed statistical overrepresentation test for these genes. According to the value of fold enrichment, the highest one was negative regulation of viral process (fold enrichment = 2.27, *p* = 5.16e-03; Table 5). What’s more, top three categories were all related to virus regulation. In this list of top 10 categories, four of them were associated with viral process and one was in connection with immune response (neutrophil activation involved in immune response; Table 5). In addition, the most significant category was neutrophil activation (fold enrichment = 1.85, *p* = 3.76e-11; Table 5). The results for all identified GO categories with corrected *p*-value less than 0.05 from this analysis are illustrated in Supplementary Table S4.

**TABLE 5:** Top 10 identified gene ontology categories from PANTHER overrepresentation test of our eQTL analysis results, according to fold enrichment.

**Gene ontology biological process category**	**# Ref genes**	**# Genes**	**Expected**	**Fold enrichment**	***p*-value**
Negative regulation of viral process (GO:0048525)	99	46	20.25	2.27	5.16e-03
Regulation of viral genome replication (GO:0045069)	94	42	19.23	2.18	3.95e-02
Regulation of viral life cycle (GO:1903900)	144	64	29.46	2.17	1.88e-04
Negative regulation of multi-organism process (GO:0043901)	180	75	36.82	2.04	1.68e-04
Regulation of symbiosis, encompassing mutualism through parasitism (GO:0043903)	231	92	47.25	1.95	3.77e-05
Regulation of viral process (GO:0050792)	202	80	41.32	1.94	4.64e-04
Neutrophil activation (GO:0042119)	496	188	101.46	1.85	3.76e-11
Neutrophil activation involved in immune response (GO:0002283)	487	184	99.62	1.85	9.77e-11
Granulocyte activation (GO:0036230)	501	189	102.49	1.84	4.86e-11
Neutrophil degranulation (GO:0043312)	483	182	98.8	1.84	1.72e-10

*# Ref genes: the number of genes in the reference list that map to this particular annotation data category. #Genes: the number of genes in our eQTL analysis identified gene list that map to this annotation data category. Expected: the number of genes we would expect in our gene list for this category, based on the reference list. Fold enrichment: # Genes/Expected. *p*-value: Bonferroni-corrected *p*-value.*

**FIGURE 4:**
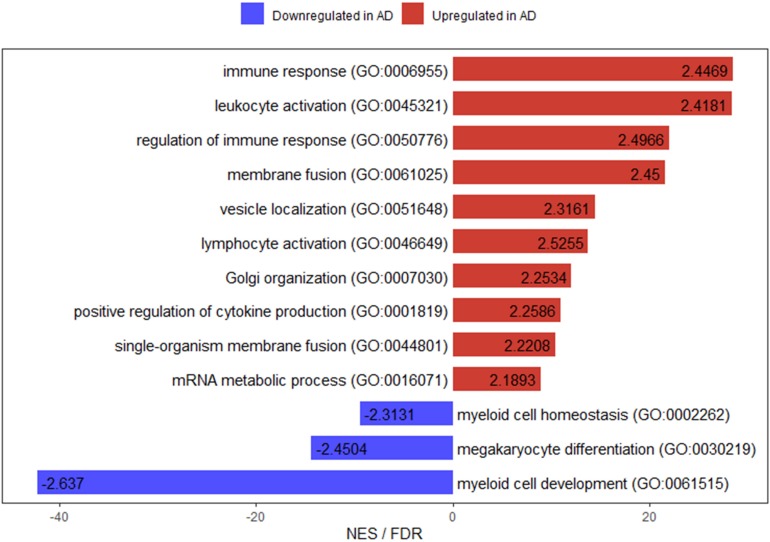
Bar plot for enriched gene ontology categories with FDR < 0.25 from GSEA of our eQTL analysis results. The numbers on the bars are the normalized enrichment scores for the corresponding gene ontology categories. NES: normalized enrichment score.

In addition to overrepresentation test, we also implemented GSEA for these over 4000 significant genes identified by our eQTL analysis. For the gene sets (GO categories) upregulated (positively enriched) in AD, 10 of them were significant at FDR < 0.25. On the other hand, there were only 3 such categories for gene sets downregulated (negatively enriched) in AD (Figure 4). The top positively enriched GO category was immune response (NES = 2.4469, *p* < 0.001, FDR = 0.0859), while the top negatively enriched gene set was myeloid cell development (NES = −2.6369, *p* < 0.001, FDR = 0.0624; Figure 4). There was another GO category related to immune response (regulation of immune response, NES = 2.4966, *p* < 0.001, FDR = 0.1136) in the top 10 significant positively enriched gene sets, which ranked third. Moreover, two of the significant downregulated gene sets were for myeloid cells. The full lists for upregulated and downregulated results with *p* < 0.05 of GSEA are in Supplementary Tables S5, S6, respectively.

## Discussion

In 2014, a study revealed a significant reduction of CSF sTREM2 in AD patients (*n* = 56) compared to control individuals (*n* = 88, *p* = 0.001; Kleinberger et al., 2014). However, 2 years later, another study reported that the CSF sTREM2 levels were significant higher in AD cases compared to controls [median and range: 1028 (244–2570) and 832 (163–2196) pg/ml, respectively; *p* = 0.015] in the Knight Alzheimer’s Disease Research Center (ADRC) cohort with 73 AD subjects and 107 controls (Piccio et al., 2016). Here, we detected that the difference of CSF sTREM2 levels between AD and normal participants was not significant in ADNI cohort (Figure 1A). These inconsistent results from different cohort studies may be caused by the timing for measuring the CSF sTREM2 levels in AD patients. Some studies for different cohorts report that the CSF sTREM2 increased in early symptomatic stages of AD, but it decreased in late stage of the disease (Heslegrave et al., 2016; Suárez-Calvet et al., 2016b; Liu D. et al., 2018). The mechanisms behind such findings still remain elusive. A possible reason would be that a part of sTREM2 released from microglia is retained within the barrier formed by microglia around plaques in the late stage of AD (Condello et al., 2015; Wang et al., 2016). Longitudinal data is required for further study about the relation between CSF sTREM2 levels and disease states. Additionally, another explanation for the inconsistency would be the different proportions of subjects with TREM2 variants in their studies. Some TREM2 variants affect CSF sTREM2 levels dramatically (Piccio et al., 2016; Wolfe et al., 2018).

CSF total tau and p-tau are passively released into CSF by dying neurons. CSF total tau levels reflect the intensity of neuronal damage and degeneration, with higher amounts of tau releasing to CSF for more intense neurodegeneration. Similar to total tau, some studies report correlation between high CSF p-tau and higher rate of cognitive decline (Blennow et al., 2010). We showed that CSF sTREM2 levels were positively correlated with CSF total tau and phosphorylated tau levels for all disease status in ADNI cohort (Figures 1D,E), which were also reported by some previous studies (Heslegrave et al., 2016; Piccio et al., 2016). The mechanism for CSF sTREM2 in pathogenesis of AD is still unclear based on these preliminary findings. Further studies are needed to reveal its role in neurodegeneration. As for CSF Aβ_42_, some studies showed that its levels were not correlated with CSF sTREM2 level generally (Heslegrave et al., 2016; Piccio et al., 2016). However, our analyses by disease stages detected that there were positive correlations between them for all status except for EMCI group in ADNI. Furthermore, the correlation was strongest for SMC group (Figure 1C). These imply CSF sTREM2 may play a crucial role at the very early symptomatic stage of AD.

Our QTL analysis showed that rs7232 was the most significant SNP associated with CSF sTREM2 level. A recent study for Chinese Alzheimer’s Biomarker and Lifestyle (CABLE) cohort (Hou et al., 2019) also reported this association was significant (*p* = 0.00106). Moreover, one study showed that rs7232 correlated with atrophy rate of left middle temporal and minor T allele carriers had less loss in the volume of left middle temporal than A allele homozygotes subjects (Ma et al., 2016). In addition, rs7232 was identified as a protective locus for AD (Lambert et al., 2013) in combined dataset of International Genomics of Alzheimer’s Project (IGAP) stages 1 and 2 (OR: 0.90, 95%CI: 0.87–0.92, *p* = 2.621 × 10^–14^). Our eQTL analysis detected that rs7232 was significantly associated with its located gene MS4A6A, which was supported by other studies (Proitsi et al., 2014). Membrane Spanning 4-Domains A6A (MS4A6A) has been identified as one of the significantly associated loci with AD (Hollingworth et al., 2011; Deng et al., 2012). MS4A6A expression levels were found to be associated with elevated Braak tangle and Braak plaque scores (Karch et al., 2012). Furthermore, one study reported that MS4A6A expression was significantly correlated to AD-related neurofibrillary pathology and tau phosphorylation (Martiskainen et al., 2015). Additionally, the expression of MS4A6A is highly correlated with the expression of TREM2 in the brain (Hou et al., 2019). For both MS4A6A and TREM2 are mainly expressed on microglia cells in the brain (Darmanis et al., 2015), we speculate that MS4A6A may regulate TREM2 expression and the levels of CSF sTREM2. The second most significant SNP associated with CSF sTREM2 was rs1582763 from our GWAS (Table 2), which was also highly associated with MS4A6A gene expression according to our eQTL analysis. These associations were justified by studies from other cohorts (Deming et al., 2018). Similar to rs7232, rs1582763 was identified to be associated with reduced AD risk (Jun et al., 2016). Our GWAS demonstrated that the top significant SNPs associated with CSF sTREM2 levels were located within or near the MS4A gene cluster and the genes of top significant SNP-gene pairs were also from MS4A gene family (Tables 2, 3). The MS4A gene cluster encodes cell membrane proteins. In addition to possibly being involved in the regulation of calcium signaling, MS4A gene cluster has also been reported to be involved in immune-system function (Ma et al., 2015). More recently, a study showed that MS4A4A co-localized with TREM2 in human macrophages and both proteins were upregulated in response to IL-4-mediated stimulation (Deming et al., 2019). They also found that antibody-mediated targeting of MS4A4A was sufficient to reduce sTREM2 levels in human macrophages. All these provide strong evidence of a biological relation between TREM2 and proteins in the MS4A gene cluster. Additional studies are required to understand the mechanisms for how MS4A gene cluster modulates TREM2, and affects sTREM2 levels.

As the most significant variant associated with AD risk in TREM2 coding regions, p.R47H (rs75932628) was also genotyped in ADNI cohort. However, there are no subjects carrying its minor allele T for the available SNP data. Besides p.R47H, the minor allele (T) of TREM2 rare variant p.R62H (rs143332484) was also reported to be significantly associated with increased AD risk (Cuyvers et al., 2014). But it was not genotyped in ADNI genotype data. These may be due to TREM2 coding variants present very low frequency among people [minor allele frequency (MAF) < 0.5%]. Furthermore, some other TREM2 variants have been identified to be associated with AD status (Jin et al., 2014; Guerreiro et al., 2013), including p.T96K (rs2234253), p.H157Y (rs2234255), p.L211P (rs2234256), and p.W191X (rs2234258). Our GWAS showed that p.T96K and p.L211P were associated with CSF sTREM2 levels (*p* = 0.005946, 0.009005, respectively; Supplementary Table S1), while p.H157Y and p.W191X were not as a result of their extreme low MAF. These imply that TREM2 variants may modify CSF sTREM2 levels.

For the pathway analyses, we found that some biological processes related to virus regulation were significantly enriched from our statistical overrepresentation test (Table 5). Interestingly, a previous study for a different AD cohort reported similar results (Piccio et al., 2016), which identified that the top two significant GO categories were receptor-mediated endocytosis of virus by host cell and endocytosis involved in viral entry into host cell (both with fold enrichment > 5 and *p* = 4.79e-06). On the other hand, our GSEA showed that some categories for immune response were significantly positively enriched (Figure 4). What’s more, our previous study (Liu C. et al., 2018) illustrated that how poliovirus receptor-related 2 (PVRL2), a gene located near the APOE locus and mediating the entry of herpes simplex virus (HSV), contributed to the progression of AD. Additionally, some studies reported that the accumulation of Aβ plaque deposits might be a consequence of the over-production of Aβ peptide during viral infection of the brain and Aβ peptide acted as a defense molecule of the innate immunity (Porcellini et al., 2010; Kumar et al., 2016). The viral hypothesis for AD development has been proposed almost immediately after the first case of AD was reported by Alois Alzheimer in 1907 (Lawrence, 2017). Researchers started to focus on the herpes simplex virus type 1 (HSV1) at the beginning of 1980s (Ball, 1982; Gannicliffe et al., 1986). So far, more than 100 studies have connected AD with some forms of pathogen (Itzhaki et al., 2016). People may argue that, if viruses do play a causal role in AD, then why there are so many individuals who are infected with the viruses but without developing AD? This may be due to the feature of many types of virus and bacteria that it is possible to be infected but without being affected. In recent studies, an important issue about the association of viruses with the substantial accumulation of Aβ in subjects who are cognitively normal is still not addressed (Eimer et al., 2018; Readhead et al., 2018). The researchers suggest that it may be the case that Aβ is protective in such cases of participants (Balin and Hudson, 2018; Eimer et al., 2018). If viruses or other microbes contribute to the development of AD, people may reason that could antiviral drug inhibit AD progression. Some research illustrate that antivirals have such effect. In one study, researchers found that three different antiviral agents reduced Aβ and p-tau accumulation for Vero cell cultures infected with HSV1 (Wozniak et al., 2011). A recent nationwide cohort study from Taiwan provides the first population-level evidence. This study enrolled 33,000 subjects that about one-quarter of whom were newly diagnosed with HSV infection and followed them for 15 years. The researchers found that subjects with HSV infection showed 2.5 times more likely to develop dementia than those without infection. More importantly, compared with HSV infected participants without receiving therapy, subjects with HSV who were treated with antivirals had a 10-fold reduction in the risk of dementia development (Tzeng et al., 2018). These studies show a potential causal role for viruses in AD. However, the exact nature of the link between viruses and AD is still ambiguous. More clinical trials of antiviral drugs are needed to evaluate their impacts on AD.

## Conclusion

We started from CSF sTREM2 levels to explore the pathogenesis of AD according to the data in ADNI cohort. Firstly, we checked the CSF sTREM2 levels by disease status and found that there was no significant difference between AD and NL groups, though they were elevated with disease stages. Next, we studied the correlation between CSF sTREM2 levels and other AD highly associated CSF and clinical biomarkers, such as CSF tau, p-tau, Aβ_42_ levels, ADAS13 scores and hippocampus volumes, demonstrating that CSF sTREM2 levels were significantly positively correlated with CSF total tau and phosphorylated-tau levels for all disease status. Furthermore, we performed QTL analysis by setting CSF sTREM2 levels as the phenotype and identified that the SNPs located within or near the MS4A gene cluster were significantly associated with them, with rs7232 and rs1582763 as the top two significant SNPs. After that, our eQTL analysis for these significant SNPs showed that they were also associated with the expression of the genes from MS4A gene family. Additionally, our pathway analyses for the important genes from the results of GWAS illustrated that the biological processes for virus regulation and immune response were highly associated with AD. According to our study, we speculated that the genetic architecture of AD patients might increase their susceptibility to viral infections of the brain. The more frequent immune activation and response against viral infections may result in progressive neurodegeneration and lead to AD finally.

## Data Availability Statement

Publicly available datasets were analyzed in this study. This data can be found here: http://adni.loni.usc.edu.

## Author Contributions

CL analyzed the data and wrote the manuscript. JY helped with the interpretation of the results, writing of the manuscript, the study design, and statistical analysis. Both authors oversaw the overall research plan, the study design, and statistical analysis, and read and approved the final manuscript.

## Conflict of Interest

The authors declare that the research was conducted in the absence of any commercial or financial relationships that could be construed as a potential conflict of interest.

## Footnotes

1 http://adni.loni.usc.edu

2 http://www.adni-info.org

3 http://www.affymetrix.com

4 http://locuszoom.org

5 http://pantherdb.org

6 http://www.geneontology.org

7 http://software.broadinstitute.org/gsea/index.jsp
