# Supplementary figures and images for: Genome-Wide Association Studies for Cerebrospinal Fluid Soluble TREM2 in Alzheimer’s Disease
Source: Front Aging Neurosci. 2019 Oct 25;11:297. doi: 10.3389/fnagi.2019.00297 (PMC6823606; doi:10.3389/fnagi.2019.00297)

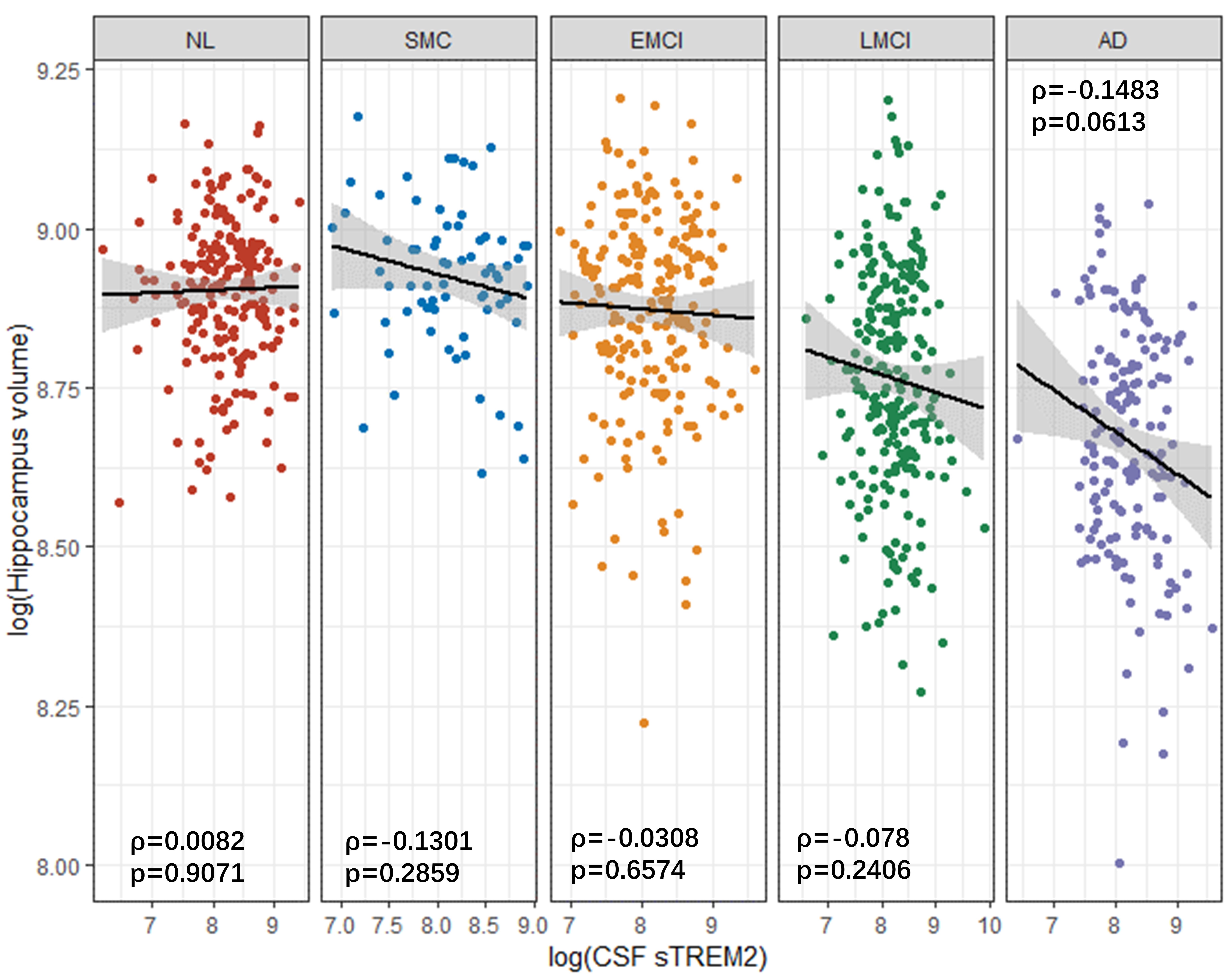

Supplement: Supplementary file 1 [file Image_1.JPEG]
