# Supplementary material for: Genome-Wide Association Studies for Cerebrospinal Fluid Soluble TREM2 in Alzheimer’s Disease
Source: Front Aging Neurosci. 2019 Oct 25;11:297. doi: 10.3389/fnagi.2019.00297 (PMC6823606; doi:10.3389/fnagi.2019.00297)
Supplement: Supplementary file 2 [file Table_1.DOCX]

Supplementary Material

Genome-Wide Association Studies for Cerebrospinal Fluid Soluble TREM2 in Alzheimer’s Disease

Changan Liu, Jun Yu

*** Correspondence:** Changan Liu: [podlca@math.uh.edu](mailto:podlca@math.uh.edu)

# Supplementary Figures and Tables

## Supplementary Figures

**Figure S1.** The correlation plot between log transformed CSF sTREM2 levels and log transformed hippocampus volumes.

## Supplementary Tables

**Table S1.** The results for all identified SNPs with p<0.05 from our QTL analysis for CSF sTREM2.

**Table S2.** The results for all identified SNP-gene pairs with p<0.05 from our cis-eQTL analysis for CSF sTREM2 related SNPs.

**Table S3.** The results for all identified SNP-gene pairs with FDR <0.05 from our trans-eQTL analysis for CSF sTREM2 related SNPs.

**Table S4.** The results for all identified gene ontology (GO) categories with corrected p-value less than 0.05 from our statistical overrepresentation test for CSF sTREM2 related genes.

**Table S5.** The results for upregulated gene ontology (GO) categories with p<0.05 from our GSEA for CSF sTREM2 related genes.

**Table S6.** The results for downregulated gene ontology (GO) categories with p<0.05 from our GSEA for CSF sTREM2 related genes.
